# AEROBIC EXERCISE ATTENUATES FRAILTY IN AGING MALE AND FEMALE C57BL/6 MICE AND EFFECTS SYSTEMIC CYTOKINES DIFFERENTIALLY BY SEX

**DOI:** 10.1093/geroni/igad104.0063

**Published:** 2023-12-21

**Authors:** Susan Howlett

**Affiliations:** Dalhousie University, Halifax, Nova Scotia, Canada

## Abstract

DOI: 10.1093/gerona/glab297

